# Alginate genes are required for optimal soil colonization and persistence by *Pseudomonas fluorescens* Pf0-1

**DOI:** 10.1099/acmi.0.000021

**Published:** 2019-05-03

**Authors:** Douglas C. Marshall, Brianna E. Arruda, Mark W. Silby

**Affiliations:** 1 Department of Biology, University of Massachusetts Dartmouth, 285 Old Westport Road, North Dartmouth, MA 02747, USA

**Keywords:** *Pseudomonas fluorescens*, biological control, water stress, soil colonization

## Abstract

*
Pseudomonas fluorescens* strains are important candidates for use as biological control agents to reduce fungal diseases on crop plants. To understand the ecological success of these bacteria and for successful and stable biological control, determination of how these bacteria colonize and persist in soil environments is critical. Here we show that *P. fluorescens* Pf0-1 is negatively impacted by reduced water availability in soil, but adapts and persists. A pilot transcriptomic study of Pf0-1 colonizing moist and dehydrated soil was used to identify candidate genetic loci, which could play a role in the adaptation to dehydration. Genes predicted to specify alginate production were identified and chosen for functional evaluation. Using deletion mutants, predicted alginate biosynthesis genes were shown to be important for optimal colonization of moist soil, and necessary for adaptation to reduced water availability in dried soil. Our findings extend *in vitro* studies of water stress into a more natural system and suggest alginate may be an essential extracellular product for the lifestyle of *P. fluorescens* when growing in soil.

## Introduction

Biological control of plant pathogens holds promise as an alternative to the use of chemical pesticides in horticulture [1–3], which is widely perceived as a negative aspect of disease control. The use of bacteria to control pathogens may allow a reduction in chemical applications or in some cases even the elimination of need for chemical control of pathogens. Reducing chemical pesticide use is desirable among some groups of consumers of horticultural products, and producers of organic produce. Even as part of an integrated pest management strategy on conventional farms, the addition of biological control agents may be beneficial, resulting in reduced costs to growers, and reducing dependence on chemicals to which resistance might arise.

Bacteria of the genus *Pseudomonas* are frequently found in association with plants and in bulk soil environments. Their ubiquity, abundance, and metabolic versatility suggest important ecological roles. In addition, several *P. fluorescens* strains have been studied extensively for their potential in the biological control of plant pathogens. Many *P. fluorescens* strains make potent secondary metabolites *in vitro* and have disease suppressive activity in greenhouse trials. Prominent examples include *P. protegens* (formerly *P. fluorescens *) Pf-5 [for example 4–6] and *P. fluorescens* 2–79 [for example 7, 8]. Despite much promise, biological control has met with limited success in a commercial sense because of variable results in field applications. While there is extensive knowledge on the chemistry of secondary metabolites and the genetics and genomics of their production [9, 10], the understanding of survival and persistence mechanisms is less well developed. These features are important given that rapid colonization may allow competitive exclusion of pathogens, while persistence ensures that the biological control agent is present for a sufficient period of time to be effective.

While several studies have worked toward establishing determinants of fitness in natural environments [for example 11–18], they are typically well controlled and avoid environmental fluctuation. However, in natural and agricultural settings, *P. fluorescens* is likely to encounter fluctuations in a range of biotic and abiotic conditions. The ubiquitous presence of *P. fluorescens* in soils suggests that they are well adapted to these changes, but the mechanisms by which they adapt to fluctuations while growing in soil are not well studied. An important parameter, which is subject to frequent change, is moisture content. Irrigation in agriculture means that water content of soil fluctuates, and in natural environments soil moisture will change with the incidence of precipitation. Ongoing climate change may also lead to increased drought in agriculturally important regions. Given the importance of bacteria in maintaining soil health and by extension the health of plants, understanding how bacteria respond to low moisture levels and the associated osmotic stress in soil is likely to become increasingly necessary. Several studies have addressed the physiological processes in bacteria, which are important for resisting osmotic stress [for example 19–21]. The production of exopolysaccharides was associated with protection from osmotic stress in a sand system [22], and the protective contribution of the production and accumulation of compatible solutes is well known [23]. The potential of drought-tolerant *Pseudomonas* sp. to enhance seed germination and plant growth during water stress has recently been shown [24], although the underlying mechanisms have not yet been reported. Global studies of bacterial responses to osmotic stress are largely confined to laboratory systems. Some studies of how *Pseudomonas* spp. respond to dehydration and altered osmolarity have been carried out by adding solutes to reduce water availability, or using an experimental system termed the pressurized porous surface model (PPSM) [25]. While these studies have begun to expose the potential responses to dehydration, showing widespread gene expression changes and implicating exopolysaccharides (EPS) in adaptation to water stress, they do not necessarily reflect the response that may occur in more natural settings. To build on *in vitro* studies on dehydration and osmotic stress, we undertook a study in which the response of *P. fluorescens* Pf0-1, to dehydration stress was examined while the bacteria were growing in soil. Pf0-1 is an isolate from sandy loam soil in Massachusetts, USA [26], which has served as a model in soil colonization studies [for example 14, 15, 27, 28]. Our data are consistent with a model in which *P. fluorescens* Pf0-1 responds rapidly to a decrease in moisture, and uses the secreted polysaccharide alginate as a means of adaptation to dehydration and for optimal persistence in soil under both favourably moist and dehydrated conditions.

## Methods

### Strains, plasmids and culture conditions, and primers

Bacterial strains and plasmids used in this study are listed in Table 1. Unless otherwise stated, *E. coli* strains were grown in LB (Lysogeny Broth) medium [29] at 37 °C with shaking at 150 r.p.m., while *P. fluorescens* strains were grown at 28 °C in *Pseudomonas* minimal medium (PMM) [30] with shaking at 150 r.p.m. When required, antibiotics were used at the following concentrations: 50 µg ampicillin ml^−1^; 25 µg kanamycin ml^−1^; 10 µg nalidixic acid ml^−1^; 10 µg or 25 µg tetracycline ml^−1^ (for *E. coli* or *P. fluorescens *, respectively); and 20 µg streptomycin ml^−1^. Growth media were solidified with 1.5 % (w/v) bactoagar when required. Oligonucleotide primers were synthesized by Invitrogen, and are listed in Table 2.

**Table 1.** Bacterial strains and plasmids

Strains and plasmids	Genotype or description	Reference or source
***E. coli***		
DH5αλ pir	φ80d*lacZ*ΔM15 Δ(*lacZYA-argF*)*U169 recA1 endA1 gyrA96 thi-1 hsdR17 supE44 relA1 deoR* _λ*pir*	[31]
S17-1	*recA pro hsdR* RP4-2-Tc::Mu-Km::Tn*7* λ-*pir*	[32]
***P. fluorescens***		
Pf0-1	Wild-type, Ap^r^	[26]
Pf0-1Δ*algB*	Δ*algB* (bases 49462–50781 removed from Pf0-1)	This study
Pf0-1Δ*algD*	Δ*algD* (bases 1119622–1120929 removed from Pf0-1)	This study
Pf0-1Δ*alg44*	Δ*alg44* (bases 1116871–1117999 removed from Pf0-1)	This study
**Plasmids**		
pGEM-T Easy	Ap^r^; cloning vector for PCR products	Promega
pSR47s	Km^r^; *sacB*-containing suicide vector (requiring R6K replication origin)	[33]

Ap^r^, ampicillin resistance;Km^r^, kanamycin resistance.

**Table 2.** Oligonucleotide primers synthesized for this study

Primers	Sequence (5′−3′)
algB-D5f	tgcatatctgcccgggctga
algB-D5r	catcggcggatgctcacagtgctcagtggctgattcc
algB-D3f	ggaatcagccactgagcactgtgagcatccgccgatg
algB-D3r	gcgtgctggaccgaattg
algD-D5f	ccagcagctcatcggacat
algD-D5r	gaggtaattgcgatgcgctaacgcagcagcaagcct
algD-D3f	aggcttgctgctgcgttagcgcatcgcaattacctc
algD-D3r	gcatagctcataccaccgg
alg44-D5f	tacagcagcacctgagcg
alg44-D5r	tgaataccgccgtcaacgaagccatggcagccggtct
alg44-D3f	agaccggctgccatggcttcgttgacggcggtattca
alg44-D3r	tgttccgctggtacggca

### Soil growth assays

The soil used was a fine loam from Sherborn, Massachusetts, sterilized by gamma irradiation. The chemical and physical characteristics of this soil have been described [34]. Bacterial strains were grown for 16 h in PMM with appropriate antibiotics. Cells were then diluted in sterile, distilled H_2_O (sdH_2_O) to approximately 1×10^5^ c.f.u. ml^−1^. The soil growth assay was carried out as described previously [14] but with the addition of 0.5 % (w/w) CaCO_3_ to increase the pH of the soil to approximately seven, as was done previously [15]. Briefly, 1 ml of the diluted cell suspension was added to 5 g of soil and mixed well. The added liquid volume achieved a water-holding capacity of approximately 50 %, after which the inoculated soil samples were transferred to 15 ml conical polypropylene tubes. The bacteria were allowed to acclimate for 30 min, after which a 0.5 g sample of soil was removed and bacteria were recovered and enumerated as has been described previously [28]. The 30 min sample was treated as the initial recoverable population. Samples (0.5 g) were taken periodically for up to 10 days post-inoculation, and culturable bacterial populations were determined from these. Between sampling points, the soil was kept in the dark at room temperature (approximately 22 °C). This procedure was used for both moist and dried (see below) soil.

### Dehydration of soil

Bacteria were allowed to colonize soil for 48 h prior to any dehydration treatment. After the 48 h period, soil samples were placed into a sterile petri dish on a balance, and left open until the desired volume of water had evaporated from the sample. Reduction in water content was determined by loss of weight, based on the 1 g of water used in the initial inoculation. In the initial experiments 0.2, 0.4 and 0.6 g of water were lost by evaporation. Since 1 ml of liquid represents 50 % of water-holding capacity (WHC), 0.2 ml of liquid would represent 10 % WHC. Thus the loss of 0.2 g of liquid weight is equivalent to a reduction to 40 % of WHC. Likewise, loss of 0.4 g and 0.6 g equate to final WHC of 30 and 20 %, respectively. The pilot transcriptome experiment and experiments on the fitness of alginate mutants used soil that was dried to 30 % WHC. After dehydration, bacterial samples for c.f.u. determination were taken immediately, and then periodically after.

### DNA manipulation and Sanger sequencing

DNA manipulations were carried out according to standard protocols [35] or as recommended by the enzyme or kit manufacturer. Restriction and DNA modification enzymes were purchased from New England Biolabs (Ipswich, MA), Invitrogen (Carlsbad, CA) and Agilent (Santa Clara, CA). Genomic DNA for PCR amplification of DNA to be cloned was purified using the Promega Wizard DNA Isolation Kit, and amplified using Herculase II Fusion DNA polymerase (Agilent). Where appropriate, PCR products were A-tailed (Klenow exo-; NEB) and cloned with pGEM-T Easy (Promega; Madison, WI). Screening of mutants and for positive clones was carried out by colony PCR using *Taq* polymerase with Thermopol II buffer (NEB). Plasmid DNA was purified from *E. coli* using a QIAprep Spin Miniprep kit (Qiagen, Valencia, CA), and DNA fragments were purified from agarose gels using a QIAEX II Gel Extraction Kit (Qiagen). Sanger sequencing was carried out at the MGH DNA Core Facility (Cambridge, MA).

### Construction and characterization of *algB*, *alg44* and *algD* mutants

Deletion mutants were constructed using splicing by overlap extension (SOE)-PCR [36]. Primers used to amplify approximately 500 bp on both sides of each target gene, and to splice those fragments, are shown in Table 2. Deletion constructs generated by PCR were first cloned with pGEM-T Easy, and then released with *Not*I digestion. These constructs were then cloned with pSR47s, transferred to Pf0-1 by conjugation, and selected for by kanamycin resistance and sucrose susceptibility. Allele exchange was used to replace the wild-type sequence with the deletion. After growth of transconjugants without selection, putative recombinants lacking pSR47s were selected (sucrose tolerant) and screened by PCR for the presence of the desired deletion.

Growth of Pf0-1 and the three alginate mutants was compared in PMM and LB media. Cultures grown for 18 h were diluted 1:100 in fresh medium in a round-bottom 96-well microtitre plate (ThermoFisher). Bacteria were grown in the 96-well plates at 37 °C with periodic shaking (every 10 min) in a Thermo Scientific Varioskan flash multimode plate reader. The OD_600_ was determined every 10 min directly after shaking over the course of 24 h. Growth curves were generated based on the averages taken for each OD_600_ reading (three technical replicates for each of three independent experiments).

### RNA extraction from Pf0-1 growing in soil

RNA from Pf0-1 growing in sterile soil (2 g samples) was harvested using MO BIO Laboratories (Carlsbad, CA) RNA PowerSoil Total RNA Isolation Kit, following the instructions from the manufacturer. These initial RNA samples were further purified by two treatments with the OneStep PCR Inhibitor Removal Kit (Zymo; Irvine, CA) in order to remove PCR inhibitors that would interfere with enzymatic steps of RNA-seq library synthesis. RNA was then treated with RQ1 DNase (Promega) for 1 h at 37 °C and purified using an RNeasy Mini Kit (Qiagen). Total RNA yields were measured using a Thermo Nanodrop 2000 spectrophotometer.

### Transcriptome library synthesis and sequencing

Prior to transcriptome library preparation, the quantity of rRNA was reduced in the total RNA extracts by treatment with the Ribo-Zero rRNA Removal Kit for Gram negative bacteria (Epicentre; Madison, WI), using 1.5 µg of starting material, and following the instructions of the manufacturer. Following rRNA depletion, transcriptome libraries for Illumina sequencing were prepared using the Epicentre ScriptSeq v2 RNA-seq Library Preparation kit beginning with 50 ng of RNA. Index primers were used to amplify the libraries, enabling multiplexing of the samples (eight in one lane, four in another). The 400–500 bp region was selected from the amplified library by excision from a 1.5 % agarose gel and subsequent purification using a QIAquick Gel Extraction Kit (Qiagen).

Libraries were sequenced at the Tufts Genomics Core (Boston, MA) using the Illumina TruSeq primer to produce single-end reads on an Illumina HiSeq2000. Reads were demultiplexed based on an index read of Illumina TruSeq 6-base index sequences.

### Transcriptome data analysis

Transcriptome data were analysed using CLC Genomics Workbench v 7.5. To remove sequence reads representing rRNA from Pf0-1, which were not removed by RiboZero treatment, all reads were mapped to the rDNA sequence and those reads that did not match the rDNA sequences were retained and mapped to the Pf0-1 genome (GenBank NC_007492.2). To quantify gene expression, the RPKM (sequence reads per kb of the gene’s coding sequence, per million total reads) for each predicted gene was determined. Data from two replicates for each treatment were averaged and then experimental and control samples were compared to identify differentially expressed genes. Statistical significance of expression changes was determined using Baggerley’s test with false discovery rate (FDR) correction for which CLC Genomics Workbench uses the Benjamini and Hochberg method [37]. A third replicate was excluded because the population of bacteria in the soil was more than tenfold lower than average, suggesting a problem with the experiment, which could impact gene expression.

### Statistical analyses

All statistical analyses of soil population data were carried out using GraphPad Prism version 6.05. Statistical tests used are specified in the text.

## Results

### Survival of *P. fluorescens* Pf0-1 under dehydration stress in soil

**Fig. 1.**
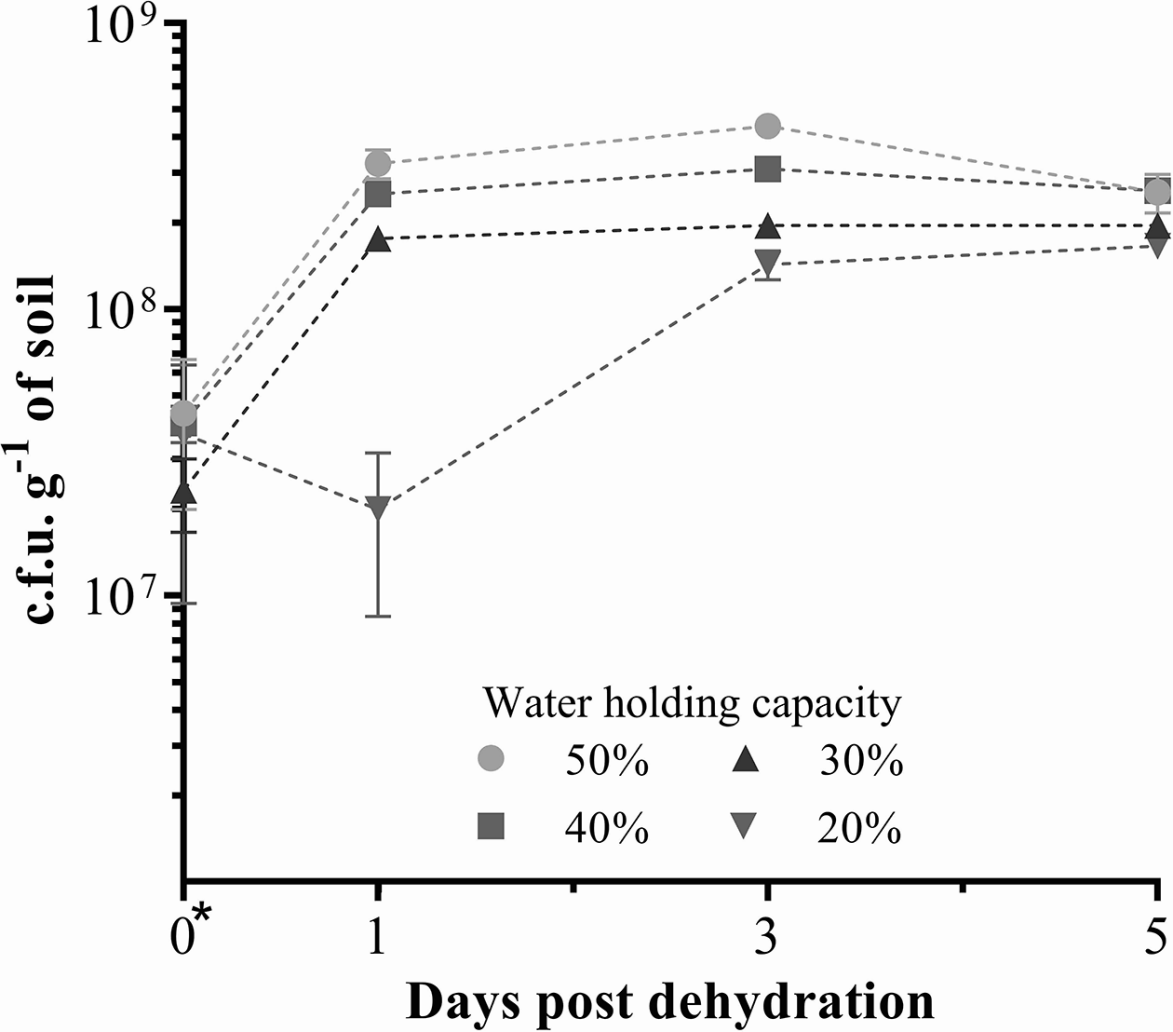
Effect of dehydration on *P. fluorescens* Pf0-1 fitness in soil. Five gram soil samples were hydrated to 50 % WHC and inoculated with Pf0-1. After 2 days, samples were dried to adjust the degree of hydration to WHC of 20, 30 and 40 %. Control samples were left at 50 % WHC. The population of Pf0-1 in experimental and control samples was determined immediately after treatment (day 0), and 1, 3 and 5 days post dehydration. Experiments were carried out at least three times. Data were analysed using one-way ANOVA with Tukey’s multiple comparison test. Error bars show standard error.

In previous studies, *P. fluorescens* Pf0-1 has been shown to persist with little population change for at least 10 days in soil moistened to 50 % WHC [for example 38, 39], and in our unpublished studies we have observed Pf0-1 populations to remain stable over the course of at least 30 days. To determine the impact of dehydration on Pf0-1 populations, we inoculated four parallel samples of moist soil with Pf0-1 and allowed the bacteria to colonize for 48 h. There was no significant difference between populations after 48 h (Fig. 1). Following colonization, three samples were dehydrated to remove 0.1, 0.2 and 0.3 g of water (leaving samples at 40, 30 and 20 % WHC), while one was left untreated (remained at 50 % WHC). The culturable population of *P. fluorescens* Pf0-1 in each treatment group was determined daily after the dehydration treatments. After 1 day, the two most severe dehydration treatments (20 and 30 % water loss) were associated with significantly lower populations than the untreated control sample (*P*<0.05), while 10 % water loss did not result in a significant difference. By 3 days post-dehydration, all three treatments resulted in significantly lower populations than the control. At day 5, the population of Pf0-1 in untreated samples and the populations in treated samples were more similar and not significantly different. The 20 % water loss condition was chosen for further study as there was a significant impact from the treatment, but sufficient viable cells remained such that extraction of nucleic acids from soil-grown samples would be feasible. Statistical significance in this section was determined by one-way ANOVA with Tukey’s multiple comparison test.

### Alginate genes respond to acute dehydration stress

Because of the technical challenges of working with RNA extracted from our sandy loam soil, and the limited power of the two replicates, we focused our analysis of the RNA-seq data on identifying candidate genes, which may have a role in resisting dehydration stress, which could be experimentally tested. We examined the set of putatively up-regulated genes from the acute dehydration sample for genes potentially important for stress tolerance. To further support the identification of candidate genes, we focused our search on clusters of genes predicted to be operons and which exhibited up-regulation of expression. We then carried out functional studies to examine the importance of these genes.

**Fig. 2.**
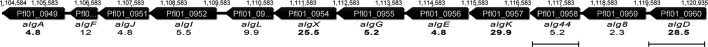
Response of the *P. fluorescens* Pf0-1 alginate biosynthetic locus to dehydration stress. Dark grey arrows show alginate genes annotated with locus tags and *alg* gene names. Genomic coordinates in the Pf0-1 genome are shown above the genes. Below each gene is the transcriptional response to dehydration expressed as fold increase in RPKM (reads per kilobase of transcript, per million mapped reads) relative to controls. Bold numbers indicate significant changes in expression. Bars under *alg44* and *algD* indicate regions that were deleted when constructing mutants.

The gene cluster *Plf01_0949–* *0960* is similar to the alginate biosynthesis cluster *PA3551-3540* of *P. aeruginosa *. From the pilot transcriptome data, half of the genes within *Plf01_0949–* *0960* displayed significantly elevated expression levels (Baggerley’s test with FDR correction using the Benjamini and Hochberg method) in response to immediate dehydration, suggesting increased alginate production (Fig. 2). Data for the other genes in the cluster suggest up-regulation but the expression change did not meet the significance threshold of *P*<0.05. The immediate response to water stress resulted in significant 4.8–29.9-fold increases in expression of six of 12 alginate genes in this locus. Expression of *algD*, *algK* and *algX*, each increased by greater than 25-fold when compared to moist control conditions. Interestingly, none of the alginate genes showed an indication of elevated expression relative to controls after long-term water stress. Because the RNA-seq used was a low power method to find candidate genes, any possible role for *alg* needed to be established by experimental means.

### Alginate is required for optimal colonization of moist soil

Although not all changes in *alg* gene expression were classified as significant by Baggerley’s test, the putative change of *alg* gene expression in response to reduced water content was intriguing in light of previous studies using culture-based simulations of reduced water content, which indicated a role for alginate or other EPS in tolerance of desiccation [40, 41]. These facts led us to explore the possible importance of *alg* genes for soil colonization and persistence.

To assess the importance of alginate for tolerance of dehydration in the soil environment, the genes *algB*, *algD* and *alg44*, were each deleted from Pf0-1. These genes were chosen because their products have distinct roles in producing alginate, and loss of each would be predicted to result in reduced ability to produce alginate. In *P. aeruginosa ,* Alg44 is necessary for alginate production, and functions as a co-polymerase for alginate polymerization [42] and interacts with the alginate polymerase Alg8 [43]. Alg44 has a PilZ domain to which cyclic di-GMP binds and promotes alginate polymerization [44]. AlgD is a cytoplasmic protein the function of which is GDP-mannose dehydrogenase for synthesis of the alginate precursor activated nucleotide sugar GDP-mannuronate [45]. AlgB in an NtrC family response regulator, which activates transcription of *algD* [46, 47], and is required for alginate production in mucoid *P. aeruginosa* isolates. Despite the relatively complex regulation of alginate production, *algB* mutants are predicted to be unable to produce alginate. We tested each deletion mutant for growth defects in laboratory culture (both PMM and LB), to rule out generalized growth defects as causes for any phenotype observed in soil. None of the *alg* deletion mutants differed from Pf0-1 during growth in either LB (doubling time approximately 37 min) or PMM (doubling time approximately 5.5 h).

**Fig. 3.**
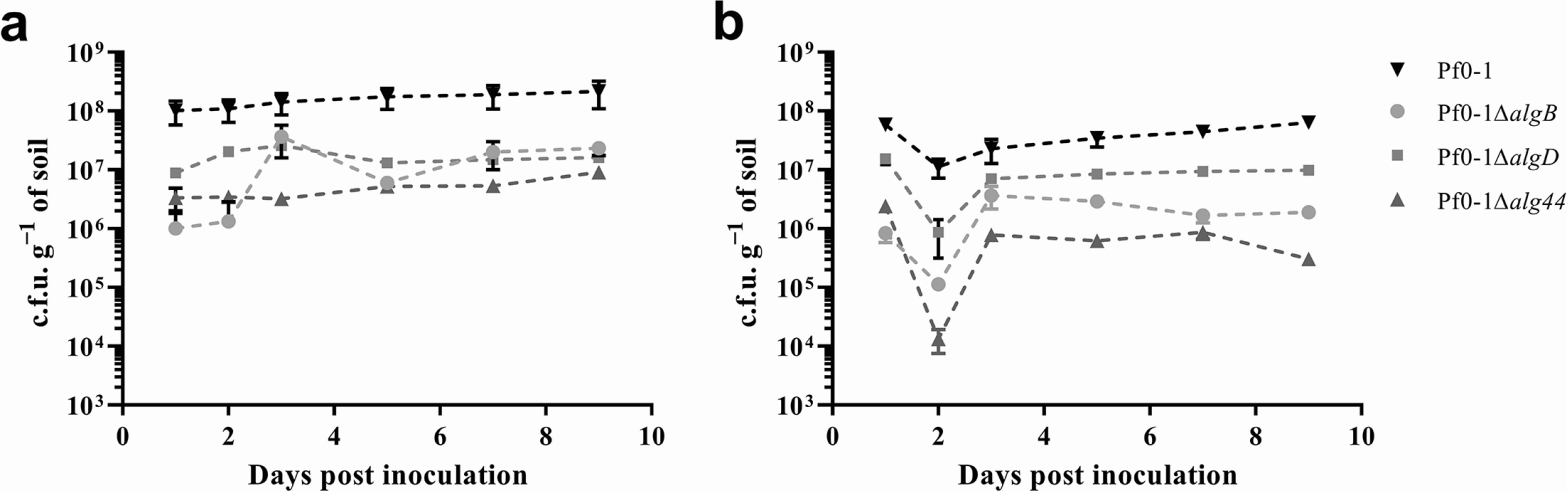
Populations of Pf0-1 and *alg* mutants in moist soil (a) and soil dried to 30 % WHC 2 days after inoculation (b). Each soil sample was inoculated with the same number of bacteria. Both panels show that each *alg* mutant was defective in colonization of moist soil (day 1 samples). Dehydration caused a decline in wild-type and mutant populations, with mutants being more dramatically affected. Experiments were carried out at least three times. Data were analysed by two-way ANOVA with post-hoc Dunnett’s multiple comparison test. Error bars show the standard error.

In moist (50 % WHC) soil conditions the Pf0-1*ΔalgB*, Pf0-1*ΔalgD* and Pf0-1*Δal*g*44* populations were each unable to establish and maintain populations as high as wild-type Pf0-1 (Fig. 3a). After 24 h of growth in moist soil, the Pf0-1*ΔalgB*, Pf0-1*ΔalgD* and Pf0-1*Δal*g44 mutant populations were at least tenfold lower than the wild-type Pf0-1, despite the soil being inoculated with approximately the same number of bacteria at the beginning of each experiment. Over the 9 days for which the population was monitored, the mutants were unable to establish the same population density as Pf0-1; their populations consistently remained at least tenfold lower. At each time point, the population of each mutant was significantly different from the wild-type (*P*<0.05, two-way ANOVA with post-hoc Dunnett’s multiple comparison test).

### Alginate is required for optimal response to dehydration of soil

To assess the importance of alginate on adaptation to dehydration stress, separate soil columns were inoculated with equal numbers of Pf0-1 and the three *alg* deletion mutants. After 1 day of colonization, mutant populations were similar to those shown above for the moist soil experiments (Fig. 3b). After 2 days, the soil was dried to 30 % WHC, after which the population of all strains tested decreased (see decline from day 1 to day 2, Fig. 3b). Of note, the three *alg* mutants each declined more than the wild-type Pf0-1 after dehydration. Pf0-1*ΔalgB* populations showed a significant sixfold greater decrease in population size following dehydration when compared to Pf0-1 dehydrated in soil (*P*=0.004, T-test), while Pf0-1*ΔalgD* populations showed a 15-fold greater decrease (not significant, *P*=0.09, T-test) when dehydrated when compared to Pf0-1 in dehydrated soil. Pf0-1*Δalg44* populations showed a significant 195-fold greater decrease in population size following dehydration when compared to Pf0-1 dehydrated in soil, the largest overall decline observed of any mutant examined in soil (*P*=0.002, T-test). At each time point, all mutants had significantly lower populations than Pf0-1 (*P*<0.05, two-way ANOVA with post-hoc Dunnett’s multiple comparison test). Each dehydrated mutant population showed growth and recovery following dehydration for the remainder of the population assay, but neither mutants nor wild-type were able to reach the population density achieved during growth in moist soil. The recovery of mutants was never sufficient to reach a population comparable to the wild-type in the dried conditions, further underscoring the importance of alginate in response to dehydration during growth in soil. After 9 days, when compared to the populations in moist soil, the populations of all alginate mutants and the wild-type strain were significantly lower than in moist soil (*P*<0.05, T-test). Dehydration clearly impacts Pf0-1, and the absence of the selected *alg* genes renders Pf0-1 less able to respond to the stress.

## Discussion

Understanding the lives of bacteria where they actually live remains challenging. Advances in DNA sequencing technologies have given unprecedented insight into the makeup and fluctuations of microbial communities in several environments including soil, but the functional characterization of the community members *in situ* is far less advanced. Conditions in soil are subject to change as a result of both abiotic and biotic perturbations, and successful members of the microbial community need to rapidly and effectively respond to these changes. To address the underlying basis of how *P. fluorescens* Pf0-1 persists in soil, and to extend research, which used *in vitro* systems, we have used a simplified sterile soil microcosm and investigated the response to one such abiotic stress, dehydration.

The population data from this study suggest that the ability of *P. fluorescens* Pf0-1 to thrive in soil is negatively impacted when the soil environment loses moisture. In the most extreme condition tested, where the water was reduced from 50 to 20 % of WHC, the population of Pf0-1 declined, while lesser reductions in moisture resulted in Pf0-1 being unable to establish the initial population maximum seen at 50 % WHC. Of note, though, is that over time the populations under all dehydration conditions tested eventually equilibrate at a similar level to that seen under the 50 % WHC condition, which we routinely consider to be favourable. These data suggest that *P. fluorescens* Pf0-1 is well adapted to fluctuations in soil moisture and the accompanying osmotic stress, and has mechanisms by which it adapts to minimize the impact of this stress.

*P. putida* responds rapidly to altered matric potential [25]. At the earliest time point (4 h after reducing matric potential) a widespread response was observed. Among the gene expression responses in *P. putida* was increased expression of alginate genes. In *Pseudomonas* sp. alginate production may represent a primary response to water stress, in addition to other functions in maintenance of a stable population. Consistent with the response of alginate genes to reduced matric potential in *P. putida *, our data indicate that alginate genes are necessary for tolerance of dehydration stress, and also for optimal soil colonization even in moist conditions. At that point, our mutant strains show that alginate was required regardless of the level of hydration. This observation may suggest that the conditions we consider to be normal in soil (50 % WHC) are not well mimicked by laboratory media, where alginate genes do not appear to be required to grow as well as the wild-type strain.

Alginate and other exopolysaccharides are important for the normal development of *P. aeruginosa* biofilms [48–50]. Loss of alginate production reduces viable count of bacteria in the biofilm, and impacts the biofilm architecture [51]. In multispecies biofilms, mutations rendering *P. aeruginosa* alginate defective or over-producing resulted in increased representation of *P. aeruginosa* in the biofilms, and over-production of alginate was not associated with increased resistance of biofilms to SDS stress [52]. Notably, under conditions of matrix stress *P. putida* produces alginate, which sequesters water into the microenvironment immediately surrounding the cells, and influences biofilm architecture [40]. In *P. fluorescens* Pf0-1, alginate could be serving similar functions facilitating long-term persistence in soil, where it is not unreasonable to suggest that the bacteria will be predominantly in a biofilm mode of growth.

Several groups have reported transcriptome experiments investigating the response to osmotic stress. These experiments have all been done under *in vitro* conditions, in contrast to our functional studies in which we used a simplified *in situ* system. Clearly, in general terms, all of these experiments have revealed the profound impact of osmotic stress on *Pseudomonas* sp. Gülez *et al*. used microarray experiments to assess the transcriptional response of *P. putida* KT2440 exposed to matric stress [25]. This study is notable in that rather than adding solutes to increase osmotic stress, the PPSM was used to specifically reduce matric potential. Gene expression profiles from the PPSM were different to those observed after adding the solute polyethylene glycol 8000, suggesting that methods, which do not add solutes, give a distinct reflection of the response. The different responses in the distinct *in vitro* systems highlight the influence of the experimental model on outcomes. While soil remains a challenging environment in which to conduct experiments, the impact of experimental model systems on the data supports our approach, which was to simulate as much as possible the natural response to lowered water availability *in situ*. Rather than adding a specific solute we reduced water availability and effectively increased the osmotic stress caused by the naturally occurring (uncharacterized) mix of compounds, which would deliver osmotic stress. The approach was limited in that it proved extremely difficult to carry out RNA-seq experiments, but by combining a pilot RNA-seq with functional studies, we have been able to use our soil system to identify the genes necessary for the response to soil moisture loss.

The ability to adapt to stressful conditions in soil is critical to the survival and persistence of the resident bacteria. We have extended the analysis of how *Pseudomonas* sp. survive reduction in water by carrying out experiments in a soil system, rather than in laboratory culture. Although our transcriptome experiments lack the level of replication necessary to survey the global gene expression response to dehydration, these data were useful in identifying candidate loci of importance in adapting to water stress. More broadly, this work shows that a limited RNA-seq experiment can be used in a similar way to *in vivo* expression technology [53] and transposon mutagenesis, to highlight potentially interesting candidate loci for further analysis. The alginate biosynthetic locus showed indications of increased gene expression after water content was reduced, suggesting a role in adaptation. Genetic analysis confirmed the importance of alginate genes in response to dehydration, and also revealed a role for alginate in persistence in moist soil. These experiments add to the understanding of factors affecting fitness in soil, which is critical for understanding microbiome communities and for the use of soil organisms in applications such as biological control.

## References

[1] Weller DM (2007). *Pseudomonas* biocontrol agents of soilborne pathogens: looking back over 30 years. Phytopathology.

[2] Fravel DR (2005). Commercialization and implementation of biocontrol. Annu Rev Phytopathol.

[3] Chet I, Inbar J (1994). Biological control of fungal pathogens. Appl Biochem Biotechnol.

[4] Rodriguez F, Pfender WF (1997). Antibiosis and antagonism of *Sclerotinia homoeocarpa* and *Drechslera poae* by *Pseudomonas fluorescens* Pf-5 *in vitro* and *in planta*. Phytopathology.

[5] Loper JE, Gross H (2007). Genomic analysis of antifungal metabolite production by *Pseudomonas fluorescens* Pf-5. Eur J Plant Pathol.

[6] Kraus J, Loper JE (1992). Lack of evidence for a role of antifungal metabolite production by *Pseudomonas fluorescens* Pf-5 in biological control of *Pythium* damping-off of cucumber. Phytopathology.

[7] Hamdan H, Weller DM, Thomashow LS (1991). Relative importance of fluorescent siderophores and other factors in biological control of *Gaeumannomyces graminis* var. *tritici* by *Pseudomonas fluorescens* 2-79 and M4-80R. Appl Environ Microbiol.

[8] Thomashow LS, Weller DM (1988). Role of a phenazine antibiotic from *Pseudomonas fluorescens* in biological control of *Gaeumannomyces graminis* var. tritici. J Bacteriol.

[9] Gross H, Loper JE (2009). Genomics of secondary metabolite production by *Pseudomonas* spp. Nat Prod Rep.

[10] Paterson J, Jahanshah G, Li Y, Wang Q, Mehnaz S (2017). The contribution of genome mining strategies to the understanding of active principles of PGPR strains. FEMS Microbiol Ecol.

[11] Rainey PB (1999). Adaptation of *Pseudomonas fluorescens* to the plant rhizosphere. Environ Microbiol.

[12] Gal M, Preston GM, Massey RC, Spiers AJ, Rainey PB (2003). Genes encoding a cellulosic polymer contribute toward the ecological success of *Pseudomonas fluorescens* SBW25 on plant surfaces. Mol Ecol.

[13] Silby MW, Cerdeño-Tárraga AM, Vernikos GS, Giddens SR, Jackson RW (2009). Genomic and genetic analyses of diversity and plant interactions of *Pseudomonas fluorescens*. Genome Biol.

[14] Silby MW, Nicoll JS, Levy SB (2009). Requirement of polyphosphate by *Pseudomonas fluorescens* Pf0-1 for competitive fitness and heat tolerance in laboratory media and sterile soil. Appl Environ Microbiol.

[15] Varivarn K, Champa LA, Silby MW, Robleto EA (2013). Colonization strategies of *Pseudomonas fluorescens* Pf0-1: activation of soil-specific genes important for diverse and specific environments. BMC Microbiol.

[16] Ghiglione JF, Richaume A, Philippot L, Lensi R (2002). Relative involvement of nitrate and nitrite reduction in the competitiveness of *Pseudomonas fluorescens* in the rhizosphere of maize under non-limiting nitrate conditions. FEMS Microbiol Ecol.

[17] Rediers H, Vanderleyden J, De Mot R (2009). Nitrate respiration in *Pseudomonas stutzeri* A15 and its involvement in rice and wheat root colonization. Microbiol Res.

[18] Philippot L, Clays-Josserand A, Lensi R (1995). Use of Tn5 mutants to assess the role of the dissimilatory nitrite reductase in the competitive abilities of two *Pseudomonas* strains in soil. Appl Environ Microbiol.

[19] Bojanovič K, D'Arrigo I, Long KS (2017). Global transcriptional responses to osmotic, oxidative, and imipenem stress conditions in *Pseudomonas putida*. Appl Environ Microbiol.

[20] Freeman BC, Chen C, Yu X, Nielsen L, Peterson K (2013). Physiological and transcriptional responses to osmotic stress of two *Pseudomonas syringae* strains that differ in epiphytic fitness and osmotolerance. J Bacteriol.

[21] Sledjeski DD, Gottesman S (1996). Osmotic shock induction of capsule synthesis in *Escherichia coli* K-12. J Bacteriol.

[22] Roberson EB, Firestone MK (1992). Relationship between desiccation and exopolysaccharide production in a soil *Pseudomonas* sp. Appl Environ Microbiol.

[23] Miller KJ, Wood JM (1996). Osmoadaptation by rhizosphere bacteria. Annu Rev Microbiol.

[24] Niu X, Song L, Xiao Y, Ge W (2018). Drought-tolerant plant growth-promoting rhizobacteria associated with foxtail millet in a semi-arid Agroecosystem and their potential in alleviating drought stress. Front Microbiol.

[25] Gülez G, Dechesne A, Workman CT, Smets BF (2012). Transcriptome dynamics of *Pseudomonas putida* KT2440 under water stress. Appl Environ Microbiol.

[26] Compeau G, Al-Achi BJ, Platsouka E, Levy SB (1988). Survival of rifampin-resistant mutants of *Pseudomonas fluorescens* and *Pseudomonas putida* in soil systems. Appl Environ Microbiol.

[27] Marshall B, Robleto EA, Wetzler R, Kulle P, Casaz P (2001). The *adnA* transcriptional factor affects persistence and spread of *Pseudomonas fluorescens* under natural field conditions. Appl Environ Microbiol.

[28] Silby MW, Levy SB (2004). Use of *in vivo* expression technology to identify genes important in growth and survival of *Pseudomonas fluorescens* Pf0-1 in soil: discovery of expressed sequences with novel genetic organization. J Bacteriol.

[29] Bertani G (2004). Lysogeny at mid-twentieth century: P1, P2, and other experimental systems. J Bacteriol.

[30] Kirner S, Krauss S, Sury G, Lam ST, Ligon JM (1996). The non-haem chloroperoxidase from *Pseudomonas fluorescens* and its relationship to pyrrolnitrin biosynthesis. Microbiology.

[31] Kolter R, Inuzuka M, Helinski DR (1978). Trans-complementation-dependent replication of a low molecular weight origin fragment from plasmid R6K. Cell.

[32] Simon R, Priefer U, Puhler A (1983). A broad host range mobilisation system for *in vivo* engineering: Transposon mutagenesis in gram-negative bacteria. Biotechnology.

[33] Matthews M, Roy CR (2000). Identification and subcellular localization of the *Legionella pneumophila* IcmX protein: a factor essential for establishment of a replicative organelle in eukaryotic host cells. Infect Immun.

[34] Mastropaolo MD, Silby MW, Nicoll JS, Levy SB (2012). Novel genes involved in motility and biofilm formation in *Pseudomonas fluorescens* Pf0-1. Appl Environ Microbiol.

[35] Green MR, Sambrook J (2012). Molecular Cloning: a Laboratory Manual.

[36] Horton RM, Hunt HD, Ho SN, Pullen JK, Pease LR (1989). Engineering hybrid genes without the use of restriction enzymes: gene splicing by overlap extension. Gene.

[37] Benjamini Y, Hochberg Y (1995). Controlling the false discovery rate: a practical and powerful approach to multiple testing. J R Stat Soc Series B Stat Methodol.

[38] Seaton SC, Silby MW, Levy SB (2013). Pleiotropic effects of GacA on *Pseudomonas fluorescens* Pf0-1 *in vitro* and in soil. Appl Environ Microbiol.

[39] Silby MW, Rainey PB, Levy SB (2004). IVET experiments in *Pseudomonas fluorescens* reveal cryptic promoters at loci associated with recognizable overlapping genes. Microbiology.

[40] Chang WS, van de Mortel M, Nielsen L, Nino de Guzman G, Li X (2007). Alginate production by *Pseudomonas putida* creates a hydrated microenvironment and contributes to biofilm architecture and stress tolerance under water-limiting conditions. J Bacteriol.

[41] Li X, Nielsen L, Nolan C, Halverson LJ (2010). Transient alginate gene expression by *Pseudomonas putida* biofilm residents under water-limiting conditions reflects adaptation to the local environment. Environ Microbiol.

[42] Remminghorst U, Rehm BHA (2006). Alg44, a unique protein required for alginate biosynthesis in *Pseudomonas aeruginosa*. FEBS Lett.

[43] Moradali MF, Donati I, Sims IM, Ghods S, Rehm BHA (2015). Alginate polymerization and modification are linked in *Pseudomonas aeruginosa*. MBio.

[44] Merighi M, Lee VT, Hyodo M, Hayakawa Y, Lory S (2007). The second messenger bis-(3′-5′)-cyclic-GMP and its PilZ domain-containing receptor Alg44 are required for alginate biosynthesis in *Pseudomonas aeruginosa*. Mol Microbiol.

[45] Deretic V, Gill JF, Chakrabarty AM (1987). Gene algD coding for GDPmannose dehydrogenase is transcriptionally activated in mucoid *Pseudomonas aeruginosa*. J Bacteriol.

[46] Wozniak DJ, Ohman DE (1991). *Pseudomonas aeruginosa* AlgB, a two-component response regulator of the NtrC family, is required for *algD* transcription. J Bacteriol.

[47] Goldberg JB, Dahnke T (1992). *Pseudomonas aeruginosa* AlgB, which modulates the expression of alginate, is a member of the NtrC subclass of prokaryotic regulators. Mol Microbiol.

[48] Chew SC, Kundukad B, Seviour T, van der Maarel JRC, Yang L (2014). Dynamic remodeling of microbial biofilms by functionally distinct exopolysaccharides. MBio.

[49] Colvin KM, Irie Y, Tart CS, Urbano R, Whitney JC (2012). The Pel and Psl polysaccharides provide *Pseudomonas aeruginosa* structural redundancy within the biofilm matrix. Environ Microbiol.

[50] Stapper AP, Narasimhan G, Ohman DE, Barakat J, Hentzer M (2004). Alginate production affects *Pseudomonas aeruginosa* biofilm development and architecture, but is not essential for biofilm formation. J Med Microbiol.

[51] Ghafoor A, Hay ID, Rehm BHA (2011). Role of exopolysaccharides in *Pseudomonas aeruginosa* biofilm formation and architecture. Appl Environ Microbiol.

[52] Periasamy S, Nair HAS, Lee KWK, Ong J, Goh JQJ (2015). *Pseudomonas aeruginosa* PAO1 exopolysaccharides are important for mixed species biofilm community development and stress tolerance. Front Microbiol.

[53] Slauch JM, Mahan MJ, Mekalanos JJ (1994). *In vivo* expression technology for selection of bacterial genes specifically induced in host tissues. Methods Enzymol.

